# Diagnostic Value of C-reactive Protein and Interleukin-8 in Risk Stratification of Febrile Neutropenic Children with Allogeneic Hematopoietic Stem Cell Transplantation

**DOI:** 10.1038/s41598-020-59814-6

**Published:** 2020-02-19

**Authors:** Tang-Her Jaing, Chih-Chen Chang, Tsung-Yen Chang, Shih-Hsiang Chen, Yu-Chuan Wen, Pei-Kwei Tsay

**Affiliations:** 1 Division of Hematology/Oncology, Department of Pediatrics, Chang Gung Children’s Hospital, Chang Gung University, Linkou, Taoyuan Taiwan; 2 Department of Medical Imaging and Intervention, Chang Gung Memorial Hospital, Linkou, Taoyuan Taiwan; 3 Department of Nursing, Chang Gung Memorial Hospital, Linkou, Taoyuan Taiwan; 4 Department of Public Health and Center of Biostatistics, College of Medicine, Chang Gung University, Taoyuan, Taiwan

**Keywords:** Chemotherapy, Cancer therapy

## Abstract

In this analysis, the levels of CRP and IL-8 were employed as a guide for designing the duration of antibiotics administration in the condition of febrile neutropenia. The importance of laboratory biomarkers is in the early diagnosis of critical illness and adjustment of further management. IL-8 is a useful biomarker for the early identification of critically ill patients, compared to CRP in FN.

## Introduction

Achievement of successful transplantation is hampered by high risk for sepsis. Hypothetically, early prediction of the severity of sepsis can lead to prompt intensive treatment resulting in improved outcome.

We analyzed the diagnostic value of serum interleukin-8 (IL-8) in predicting sepsis by examining the safety and feasibility of antibiotic de-escalation in pediatric transplant patients. A total of 30 children (median age, 7.2 years) who underwent HSCT were enrolled in this single-center prospective study. Patients were divided two groups with or without apparent focus of infection.

IL-8 and CRP levels were mutually positively correlated. IL-8 relatively decreased from 72 h and the decreased was considerably earlier than in CRP in the absence of documented bacteremia or recent remote site infection. Accordingly, antibiotics might be de-escalated/discontinued. Increased IL-8 and CRP levels were associated with infectious complications in patients which underwent allogeneic hematopoietic stem cell transplantation (HSCT). Because no significant elevations were observed among patients with graft-versus-host disease, our findings suggest that IL-8 levels in HSCT are a reliable tool for the prediction and diagnosis of infectious complications.

## Methods

Pediatric transplant patients with fever and suspected chemotherapy-induced neutropenia were consecutively evaluated for inclusion in the study. Fever was invariably defined as a single body temperature >38.5 °C, or ≥2 recordings of a temperature >38.0 °C during a 6-h period. Neutropenia was defined as absolute neutrophil count (ANC) < 0.5 × 10^9^/L. Prior treatment with antibiotics other than the usual prophylactic antibiotic treatment strategies (i.e. *Pneumocystis jirovecii*) or had undergone allogeneic HSCT in the past 3 months was an exclusion criterion. Recipients of autologous HSCT or primary immunodeficiency were also excluded. Myeloablative conditioning regimen used before HSCT were more frequently than other preparative regimens. Data was obtained from the institutional electronic database and patient records.

All included patients underwent exploration for positive physical examination findings. Blood cultures are the standard method for diagnosis of bloodstream infections. Plasma samples were collected to measure the plasma IL-8 level upon clinical presentation with FN and again 72 h.

This single-center prospective study was undertaken in Chang Gung Children’s Hospital (Taoyuan, Taiwan) between June 2016 and June 2017. IRB approval was obtained from Chang Gung Memorial Hospital (Approval number: 201600482A3). All participants, or their legal guardians if individuals were under 18 years, provided written informed consent and complied with all relevant guidelines and regulations.

Plasma samples were taken during each FN episode at initial presentation with FN and after 72 h. Thirty patients were further divided into two groups according to the subsequent development of documented infection or clinical sepsis. Infection was deemed to be microbiologically or clinically confirmed when an etiologic microorganism was isolated from relevant cultures or when clinical presentation or radiologic signs of infections (except high body temperature and heart rate) were present, respectively. Clinical sepsis is defined as having clinical signs or positive cultures consistent with infection and/or sepsis syndrome in the absence of positive blood, urine or sputum cultures. An infectious episode was defined as a documented case of bacteremia or fungemia from a sterile site. Conversely, patients having persistent fever and radiologically documented new or progressing lesions may not have an identified microorganism. In such patients, fever is often the only sign of infection.

Patients who developed FN were empirically treated with i.v. cefazolin and amikacin, with further decision being guided by relevant cultures and antibiograms. Once empirical antibiotics are initiated, antibiotic de-escalation is an important strategy advocated to ensure the effectiveness of broad-spectrum antibiotics. All the patients were clinically assessed after 72 h of antibiotic treatment if the blood culture was negative and antibiotics were de-escalated in selected patients depending on their IL-8 levels. According to a definition analysis by Orlikowsky^1^, the threshold for IL-8 plasma concentration was set to 60 ng/L.

Vital signs pointing to potential sepsis were systolic blood pressure ≤−2 standard deviation (SD) below lower limit of normal for age and sex or heart rate and respiratory rate ≥+2 SD above upper limit of normal for age and sex. Patients at risk of invasive aspergillosis should have a baseline serum tested and should be monitored once a week for increasing galactomannan antigen levels. Levels of galactomannan antigen were determined only after a minimum of 100 days after HSCT at least once weekly by Platelia^TM^ Aspergillus enzyme-linked immunosorbent assay (Bio-Rad Laboratories, Munich, Germany).

Patients with 2 consecutive plasma IL-8 levels below the cut-off value (60 ng/L) were classified as low-risk patients. In these patients, antibiotics were de-escalated, and they were hospitalized till the neutrophil count is 1.0 × 10^9^/L or higher. Patients with increased levels of IL-8 in the first or second measurement were classified being of medium risk. If after 72 h of appropriate antibiotic treatment, patients did not have for a minimum of 24 h without any sign of infection, and persistently negative blood cultures, antibiotics were terminated 2 or 3 days after their temperatures had returned to normal. If one of the aforementioned criteria was not satisfied, antibiotics were continued according to relevant provisions for such patients.

Approximately 5 ml intravenous blood was collected in EDTA medium with a prior consent of the subjects. Plasma IL-8 concentrations were measured with solid-phase, two-site chemiluminescent immunometric assay (Immunlite 1000, Diagnostic Products Corporation, Los Angeles, CA), according to the instrument manufacturer’s instructions. The lower detection limits of the assay was set at 5 ng/L, and the result of this analysis was available within 30 min. The cut-off value was based on our previous study results and was set at 60 ng/L.

Descriptive statistics were used to describe the data. Data on episodes of FN was non-normally distributed and presented as median and range. Mann-Whitney *U* test (for unpaired samples) and Wilcoxon signed-rank test (for paired samples) were used for between-group comparisons of continuous non-normally distributed variables. A *p*-value < 0.05 was used as indicator of statistical significance. Statistical Package for the Social Sciences 16.0 (SPSS, Chicago, IL) was used for all analyses.

## Results

**Figure 1:**
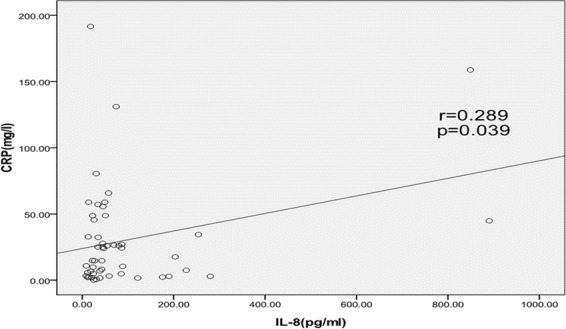
Serum interleukin-8 (IL-8) and C-reactive protein (CRP) levels were elevated and positively correlated in transplanted patients with febrile neutropenia. Liner regression; *r* = 0.289, *p* = 0.039. Each point represents data from an individual patient.

Thirty pediatric patients with FN who participated in the study were also treated and included in the efficacy analysis. Notably, no case of bacteremia was documented in the 30 patients who underwent HSCT. Children with neutropenia and febrility were classified into two groups: one with fever with apparently no explainable cause (group I, n = 29) and the other developed FN with clinically or radiologically documented new or progressing lesions (group II, n = 22) (Table 1). All recruited patients were treated empirically with broad-spectrum antibiotics as per protocol. CRP and IL-8 serum levels were mutually positively correlated in FN patients (*r = *0.289, *p* = 0.039) (Fig. 1). CRP levels were significantly lower in group I (*p* < 0.05) (Table 2). A higher standard deviation of IL-8 levels indicated greater variability among the patients.

**Table 1:** The sensitivity and specificity for C-reactive protein (CRP) and interleukin-8 (IL-8) for developing a serious complication at cut-off value of 40 mg/L for CRP and 60 ng/L for IL-8.

	Group I (n = 29)	Group II (n = 22)	*P*-value
**CRP**			
<40	25 (86.2%)	13 (59.1%)	<0.05
≥40	4 (13.8%)	9 (40.9%)	
**IL-8**			
<60	18 (62.1%)	17 (77.3%)	0.25
≥60	11 (37.9%)	5 (22.7%)

**Table 2:** Serum levels of C-reactive protein (CRP) and interleukin-8 (IL-8) in groups I versus II.

	Group I (n = 29) Mean ± SD	Group II (n = 22) Mean ± SD	*P*-value
CRP	17.6 ± 17.4	47.65 ± 51.7	<0.05
IL-8	94.9 ± 164.7	95.4 ± 182.4	0.99

^*^P-values ≤ 0.05 are considered statistically significant.

We determined levels of IL-8 and CRP in plasma among HSCT patients and found a marginally significant overall correlation between the CRP and IL-8 levels. Overall, 91 samples (89.2%) from 30 patients were concordant, indicating that both cytokines were below or above the respective cut-off value, and 11 samples (10.8%) from the patients were discordant in the respect. IL-8 levels decreased within 72 h and considerably earlier than CRP when there has been no documented bacteremia or recent remote site infection. No case of proven or probable invasive fungal infection occurred during the observation period according to the European Organization for Research and Treatment of Cancer (EORTC) guidelines.

## Discussion

The clinical usefulness of bioindicators and biomarkers to distinguish sepsis from other causes of FN in HSCT recipients are debated in certain epidemiological situations characterized by the emergence of multidrug-resistant gram-negative bacteria^2^. Transplant-related morbidity and mortality and late effects are the major limiting factors^3^. However, there is no universally reliable method which can help differentiate between patients with FN who require antibiotics and those who do not require antibiotics^4^. Administration of appropriate and timely antibiotics is crucial to decrease mortality^5^. Delaying or withholding antibiotic treatment until the identification of focus of infection may lead to lethal consequences. In addition, it leads to shortening of the duration of therapy and even termination of therapy in a subgroup of patients.

Bacterial infection has been documented in only 20–30% of pediatric cancer patients with FN^6^. Unnecessary hospital admissions and invasive medical procedures dramatically affect quality of life of the transplanted patients. Pre-engraftment CRP levels are associated with graft-versus-host disease (GVHD) and non-relapse mortality following allogeneic HSCT^7^. IL-8 levels have been found to rise prior to the increase of CRP levels, and increased IL-8 is detectable even prior to the accession of fever^8,9^.

There is no a one-size-fits-all approach for prophylaxis. However, non-critical use of antimicrobial therapy leads to unwanted adverse effects and potential antimicrobial resistance which is associated with worse prognosis and high mortality^10^. Patients undergoing HSCT should be evaluated periodically and within the context of local microbiologic epidemiology and host risk factors. In this study, we evaluated CRP and IL-8 as biomarkers for bacterial and fungal infection in FN along with their usefulness in FN during chemoradiation-induced oropharyngeal mucositis. These biomarkers may help predict the risk of bacterial infections in FN and aid in the early initiation of therapy, help in differentiating between infectious and noninfectious inflammation, make easy the wider use of highly-specific antibiotics, shorten the duration of antibiotic use, and potentially enable the identification of specific phenotype which might increase the benefit from specific therapies even further^11^. Although bacterial resistance poses an increasing and serious threat, antimicrobial de-escalation has been proposed as a potential measure to preserve the efficacy of currently available antimicrobial therapy without compromising patient outcome. This is especially important given the relatively slow pace in developing new antibiotics.

De-escalation of antibiotic therapy is a safe, but not widely accepted strategy^12^. Current guidelines are usually overly restrictive in recommending empiric antimicrobial therapy for FN. However, there a few important points which have to taken into account in the interpretation of serum CRP and IL-8 concentrates. Synthesis and release of CRP, which is an acute phase reactant, is dependent on the interplay of pro-inflammatory cytokines which are released as a part of an inflammatory response and this decreases its specificity for detecting infections. Serum CRP raises within 24 h of infection and can predict the development of fever and severe sepsis in neutropenic patients^13^; contrariwise, a minority of septic patients may have low CRP levels. Thus, CRP evaluation may not facilitate reliable discrimination of infectious and non-infectious febrile episodes^14^. On the other hand, IL-8 levels are more specific in diagnosing bacterial infections in FN and has a higher negative predictive value for bacteremia^15^. However, it has only limited value in the decision of antibiotic cessation in high-risk population. Development of fungal infections are less likely in the early engraftment phase, and most fungal infections are observed during the early post-engraftment period and the late phase, especially due to GVHD and its treatment^16^.

This analysis has two main limitations. Considering that the study population was an observational cohort, management decisions were at the discretion of the treating physician. Second, multiple variables can influence blood culture yield, including blood culture number, volume, and frequency and selection of media^17^. A specific challenge to the cancer population is the elevation in inflammatory markers driven by the tumor and mucositis. The appropriate timing for initiating and discontinuing antibacterial prophylaxis has not yet been established. Risk stratification is a recommended staring point for managing patient with FN^18^. Overall, our results confirm the very important role of IL-8 in immunologic processes; however, not much information is available on cytokine patterns which could lead to early identification and differentiation of several adverse events.

It is imperative to overcome hurdles on when to discontinue antibiotics in patients with low-and high-risk FN. Multidrug-resistant organisms are on the rise, and both awareness of initiating the appropriate therapy at the earliest convenience and the use of broad-spectrum antimicrobial drugs are gaining importance. Sepsis requires prompt risk stratification and decisive early treatment. Biochemical analyses, including CRP and procalcitonin, cultures, antigen screening, PCR, ABG, imaging and other tests should be done in the presence of high body temperature and suspected, especially when infection with highly-pathogenic organism is suspected^19^.

The concordance between IL-8 and CRP was studied to determine the threshold of CRP corresponding to IL-8 < 60 mg/L in patients with FN to be used mainly when IL-8 is not available. In addition, our results indicate that lower antibiotic coverage may be used in this patient population, but our study directly evaluated the economic impact of streamlining antibiotic administration and was not designed to test patient safety issues. Furthermore, due to a small sample size, the present study may not have been able to accurately verify the role of IL-8 in FN. Larger prospective randomized trials are necessary to overcome these limitation.

## Conclusion

IL-8 was positively correlated to CRP in FN. Low levels of CRP and IL-8 could relate to development of unexplainable fever in patients, whereas high levels could relate to the presence and development of infectious episodes. The results of this study may be objectionable inasmuch as algorithms which are guided via IL-8 levels can limit duration of antimicrobial treatments, reduce adverse events or prevent further antimicrobial resistance.
